# IDG-ViolenceNet: A Video Violence Detection Model Integrating Identity-Aware Graphs and 3D-CNN

**DOI:** 10.3390/s25206272

**Published:** 2025-10-10

**Authors:** Hong Huang, Qingping Jiang

**Affiliations:** School of Computer Science and Engineering, Sichuan University of Science and Engineering, Yibin 644000, China; huanghong@suse.edu.cn

**Keywords:** violence detection, 3D-CNN, spatiotemporal GNN, multi-object tracking, identity-aware modeling, public safety

## Abstract

**Highlights:**

**What are the main findings?**

- The proposed method efficiently detects violent behaviors in videos with significantly improved accuracy.
- The model outperforms existing methods in both detection speed and precision.

**What is the implication of the main finding?**

- It is applicable to public safety surveillance for rapid response to violent incidents.
- It provides a new reference and methodology for optimizing violent behavior detection algorithms.

**Abstract:**

Video violence detection plays a crucial role in intelligent surveillance and public safety, yet existing methods still face challenges in modeling complex multi-person interactions. To address this, we propose IDG-ViolenceNet, a dual-stream video violence detection model that integrates identity-aware spatiotemporal graphs with three-dimensional convolutional neural networks (3D-CNN). Specifically, the model utilizes YOLOv11 for high-precision person detection and cross-frame identity tracking, constructing a dynamic spatiotemporal graph that encodes spatial proximity, temporal continuity, and individual identity information. On this basis, a GINEConv branch extracts structured interaction features, while an R3D-18 branch models local spatiotemporal patterns. The two representations are fused in a dedicated module for cross-modal feature integration. Experimental results show that IDG-ViolenceNet achieves accuracies of 97.5%, 99.5%, and 89.4% on the Hockey Fight, Movies Fight, and RWF-2000 datasets, respectively, significantly outperforming state-of-the-art methods. Additionally, ablation studies validate the contributions of key components in improving detection accuracy and robustness.

## 1. Introduction

With the increasing frequency of public safety incidents, violence detection has gradually become one of the core tasks in intelligent video surveillance systems. In crowded public spaces such as shopping malls, railway stations, and campuses, achieving real-time recognition and early warning of violent events is of great practical significance for maintaining social order and preventing potential risks. Violence, by its nature, is a type of sudden behavior that heavily depends on the interactions between individuals. Existing studies have shown that relying solely on global appearance features often fails to effectively distinguish violent from non-violent scenarios, while neglecting spatiotemporal interaction patterns among people can substantially weaken a model’s representational capacity [1]. In recent years, Graph Neural Networks (GNNs) [2] have demonstrated outstanding performance in modeling entity interactions and have been widely applied to tasks such as action recognition and group behavior modeling [3]. Among these approaches, human-centered graph modeling methods [4] have attracted considerable attention from the research community. In such methods, detected individuals in videos are treated as graph nodes, and edges are constructed based on spatial proximity or interactive relationships, thereby effectively enhancing the modeling of local interaction patterns [5].

Although traditional video action recognition methods—primarily relying on handcrafted feature extraction or two-dimensional convolutional neural networks (2D-CNNs)—achieved certain progress in early studies, they inherently struggle to model dynamic interactions and long-range temporal dependencies. When confronted with real-world challenges such as complex backgrounds, target occlusion, and motion blur, their robustness and recognition accuracy are often severely constrained. Existing approaches generally suffer from two major limitations: (1) most interaction graphs are constructed on a single-frame basis, making it difficult to capture the behavioral continuity of the same individual across temporal frames [6], and (2) commonly adopted graph structures often exist in the form of “static snapshots,” lacking effective modeling of motion trajectories and identity information, which restricts the depth and completeness of temporal semantic representation [7].

To address the aforementioned challenges, this paper proposes a dual-stream violence detection framework that integrates a three-dimensional convolutional neural network (3D-CNN) [8] with an identity-aware graph neural network, termed IDG-ViolenceNet (Identity-aware Graph and 3D-CNN Fusion for Violence Detection). The core ideas of this method include

(1) Identity-Aware Graph Construction: Human instances are first detected in video frames using a YOLO-based model. A cross-frame matching algorithm then assigns unique identity labels to each instance. On this basis, a dynamic graph structure is constructed that encodes not only identity and spatial proximity but also temporal continuity and motion trajectory consistency.
(2) Dual-Branch Feature Extraction with 3D-CNN and GNN: The 3D-CNN branch captures spatial features and short-term temporal dependencies from frame sequences, while the GNN branch aggregates interaction patterns among nodes within the graph. This complementary modeling approach integrates visual features with structural interaction features.
(3) Cross-Modal Fusion Mechanism: Features from the two modalities are effectively fused and passed through a classifier for final prediction, enabling accurate recognition of violent behaviors. This design is particularly robust and adaptive in complex video scenarios involving multiple participants and intense interactions.

In practical application scenarios, IDG-ViolenceNet exhibits strong scalability and is oriented toward deployable use. It can be integrated as a front-end early-warning module into existing video surveillance platforms to help security systems proactively identify violent incidents, thereby reducing the costs and risks of post-event intervention. To validate the effectiveness of the method, we conducted experiments on three public datasets—HockeyFight, MoviesFight, and RWF-2000—achieving accuracies of 97.5% and 99.5% on HockeyFight and MoviesFight, respectively, and 89.4% on the more challenging RWF-2000. In addition, ablation studies show that the identity-aware graph construction mechanism and the cross-modal fusion strategy play a key role in performance gains, supporting the soundness and effectiveness of the proposed design.

## 2. Related Work

In recent years, video violence detection has emerged as a central research task in intelligent surveillance systems and has gradually evolved into a key research hotspot within the field of computer vision. Existing approaches can be broadly categorized into three groups: (1) handcrafted feature-based method [9], which primarily rely on manually designed spatiotemporal descriptors to capture motion patterns; (2) deep convolutional neural network (CNN)-based methods [10], which leverage end-to-end feature learning to model local appearance and temporal information; (3) graph neural network (GNN)-based and graph modeling methods [11], which emphasize the representation and reasoning of multi-object interaction relationships.

### 2.1. Handcrafted Feature-Based Methods for Video Violence Detection

Early studies on video violence recognition primarily relied on handcrafted features. These methods typically modeled violent behaviors by capturing local motion, appearance variations, and spatial structural information in videos. The core idea was to extract descriptors that reflect motion patterns—such as interest point trajectories, optical flow information, and edge gradient variations—and use them as inputs to traditional classifiers (e.g., SVM [12], Random Forest [13], or AdaBoost [14]). For instance, Hassner et al. [15] proposed a method based on MoSIFT (Motion SIFT) features, which combine SIFT spatial descriptors with local optical flow orientations to capture salient motion changes in violent events. This approach demonstrated good performance in short-term violence detection but struggled with occlusions and low-resolution scenarios. Similarly, Deniz et al. [16] employed the Histogram of Oriented Optical Flow (HOOF) as a descriptor of rapid motion in videos, achieving 90.2% accuracy on the Hockey Fight dataset. While this method was sensitive to intense actions, it was prone to false detections under background noise. Overall, handcrafted feature-based methods offer strong interpretability and simplicity of implementation, and they can achieve relatively high accuracy in constrained scenarios. However, they are inherently limited by their reliance on prior assumptions in feature design. This reliance restricts their ability to effectively capture complex multi-person interactions, which are common in violence detection scenarios. Moreover, their performance is often unstable in real-world environments, where challenges such as illumination variations, scale changes, and camera jitter are prevalent. While these methods perform well in controlled settings, their generalization capability is limited. Consequently, there has been a shift toward end-to-end deep learning approaches, particularly three-dimensional convolutional neural networks (3D-CNNs), which offer improved spatiotemporal modeling capabilities. These deep learning models, unlike handcrafted feature-based approaches, are less reliant on prior assumptions and are better equipped to handle the complexity and variability of real-world data, thus becoming the dominant method for violence recognition.

### 2.2. Deep Convolutional Neural Network (CNN/3D-CNN)-Based Methods

Unlike handcrafted feature-based approaches, methods built on deep convolutional neural networks (CNNs) can automatically learn discriminative features through end-to-end training, thereby reducing dependence on manually designed priors. In video violence detection tasks, two-dimensional convolutional neural networks (2D-CNNs) are primarily employed to model spatial features of individual frames but are inherently limited in capturing temporal dynamics across frames. To overcome this limitation, researchers introduced three-dimensional convolutional neural networks (3D-CNNs) [17], which perform convolutions jointly along spatial and temporal dimensions. This enables more effective representation of motion patterns and short-term dynamics in video segments. Owing to their strong feature representation capability, 3D-CNNs have gradually become one of the mainstream approaches for violence recognition. For example, Ghosh and Chakrabart [18] proposed the Two-stream Multi-dimensional CNN (2s-MDCN), which extracts features from RGB frames and optical flow using 1D, 2D, and 3D convolutions, achieving approximately 89.7% accuracy on a large-scale violence detection benchmark while maintaining a relatively compact architecture suitable for real-time applications. Negre et al. [19] enhanced violence recognition by combining key-frame extraction strategies with 3D-CNNs, further validating the advantages of 3D-CNNs in capturing short-term motion patterns. Kavathia and Sayer [20] investigated the effect of varying the number of consecutive frames on 3D-CNN classification accuracy and found that using three-frame inputs yielded a peak validation accuracy of 94.87%. These findings highlight the strong spatiotemporal modeling ability of 3D-CNNs within short time windows. Overall, 3D-CNN-based methods demonstrate remarkable effectiveness in extracting local motion patterns and are particularly well-suited for analyzing rapid and sudden violent actions. However, despite their advantages, these methods still face inherent limitations. First, they primarily focus on spatiotemporal modeling of individual actions while neglecting the spatial relationships and interaction semantics between multiple people, which restricts their ability to interpret complex behaviors in crowded scenarios. For example, in violent events involving multiple individuals, 3D-CNNs may fail to properly capture inter-person interactions, leading to inaccurate recognition. Second, 3D-CNNs often suffer from a lack of explicit modeling of structured inter-individual interactions, which makes them prone to misclassification in cases of occlusion, overlapping actions, or visually similar behaviors. In such cases, their performance may significantly degrade, leading to false positives or false negatives. These inherent weaknesses underscore the need for complementary techniques that can account for both individual motion and complex multi-person interactions. As a result, the integration of graph-based modeling techniques, which explicitly capture inter-person interactions and structural relationships, has emerged as a critical breakthrough for advancing video violence detection research.

### 2.3. Graph Neural Network-Based Methods for Action Recognition

Graph Neural Network [21] (GNN)-based methods for action recognition model higher-order relationships between individuals by constructing spatiotemporal interaction graphs. Specifically, these approaches typically represent persons in a video as graph nodes, establish edges based on spatial proximity or temporal consistency, and apply graph convolutions to extract structured features, thereby capturing interaction patterns and group behavior semantics. Compared with CNN/3D-CNN methods that rely solely on local receptive fields, GNNs can explicitly characterize multi-person interactions and cross-temporal dependencies through multi-layer message passing mechanisms, thus offering unique advantages in dynamic scene analysis and complex behavior modeling. In particular, for video violence detection tasks, GNNs are capable of better representing conflict relationships and adversarial interactions among individuals, thereby significantly improving recognition accuracy and robustness. For example, Yang et al. [22] proposed a Multi-Scale Attention Spatiotemporal Graph Convolutional Network (MSA-STGCN) for skeleton-based violence recognition, which integrates multiple skeleton data streams (joint positions, bone vectors, motion, etc.), multi-scale spatial graph convolutions, a hybrid dilated convolution structure, and channel attention mechanisms, leading to remarkable performance improvements. Tian and Li [23] developed a violence detection method based on multi-feature fusion with GCN, where ROI regions are extracted using a DETR detector, RGB, optical flow, and audio modalities are fused, and reasoning is performed through GCN, effectively reducing scene redundancy and improving recognition performance, making it a practical solution for large-scale video datasets. Lu et al. [24] introduced STIG-Net, a video violence detection model that extracts keypoint information of individuals and constructs spatiotemporal interaction graphs, incorporating both GNN and attention mechanisms into the graph structure to comprehensively model temporal and spatial dynamics among individuals. Experimental results demonstrate that STIG-Net achieves superior accuracy and AUC across multiple public datasets, showing strong generalization and robustness, particularly in complex backgrounds and multi-person interaction scenarios.

Although the aforementioned methods have achieved notable success in general action recognition tasks, they often overlook the spatial interaction information among individuals within a video. In multi-person violent events, aggressive behaviors are typically characterized by local interaction patterns such as physical proximity and bodily conflicts, which are difficult to capture using only global frame-level features. Therefore, explicitly modeling inter-individual interaction structures has become a key issue for further improving the performance of violence detection. This necessity has driven recent efforts to introduce graph neural networks into the field, enabling more effective capture and modeling of complex behavioral relationships. However, despite their effectiveness in action recognition tasks, these methods still face significant limitations. First, they often fail to fully model the dynamic and evolving nature of interactions over time, limiting their capacity to handle long-duration and complex behaviors in video violence detection. Second, although GNNs can capture spatial relationships, they may struggle with accurately modeling the strength and temporal consistency of interactions, especially in crowded or highly dynamic scenes. These challenges underscore the need for further research into enhancing GNN-based methods to better capture and model complex spatiotemporal interactions in video violence detection tasks.

### 2.4. Innovations and Advantages of the Proposed Method

While mainstream CNN/3D-CNN-based approaches are effective in capturing local spatiotemporal features, they generally lack the ability to explicitly model inter-person spatial interaction structures, which limits their effectiveness in real-world violence detection scenarios involving multiple individuals. On the other hand, existing GNN-based methods attempt to address this limitation by constructing interaction graphs, but they often rely on single-frame static graphs and lack identity-tracking mechanisms. This results in fragmented modeling and inconsistent cross-frame interactions, which significantly hampers their performance in dynamic environments. While recent works have integrated spatiotemporal graphs with identity preservation, these approaches often face challenges in maintaining consistent identity tracking across long sequences or in complex scenarios. To address these critical challenges, we propose an identity-aware spatiotemporal graph modeling strategy, which incorporates both the temporal consistency of identities and inter-person interaction dynamics across frames. Building upon this, we develop IDG-ViolenceNet, a cross-modal violence detection framework that integrates 3D-CNNs with graph neural networks (GNNs). Specifically, by associating the trajectories of detected individuals across frames, we construct dynamic cross-frame adjacency graphs that preserve identity consistency and explicitly model inter-person interactions. Meanwhile, the 3D-CNN branch captures local spatiotemporal features, while the GNN branch aggregates structured interaction information. The complementary representations from both branches are then fused through a cross-modal integration module. Experimental results demonstrate that our method significantly outperforms existing approaches across multiple public datasets and exhibits strong practical value and scalability in real-world surveillance scenarios, particularly in environments with high-density crowds, occlusions, and complex backgrounds.

## 3. Methodology

**Figure 1:**
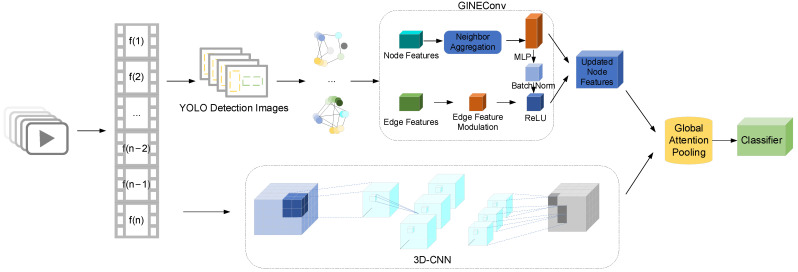
Framework of the IDG-ViolenceNet Model.

To comprehensively model the dynamic interactions among multiple individuals in videos, we propose IDG-Violence Net, a violence detection framework that integrates identity-aware graph structures with three-dimensional convolutional neural networks (3D-CNNs). The overall architecture is illustrated in Figure 1. Specifically, the input video is first divided into continuous frame sequences, and each detected person is assigned a consistent cross-frame identity ID using an object detection module combined with a lightweight tracking algorithm. Based on spatial proximity among individuals and the motion trajectories of the same identity across consecutive frames, we construct an identity-aware spatiotemporal graph to explicitly capture inter-person interactions. Subsequently, the model employs a graph neural network (GINEConv) to perform feature aggregation over the graph structure, while a 3D-CNN branch extracts spatiotemporal representations directly from the raw frame sequences. Finally, a cross-modal fusion mechanism integrates the two types of features to achieve high-precision recognition of violent behaviors. This approach preserves local action semantics while effectively strengthening the modeling of spatiotemporal interaction relationships, making it particularly suitable for real-world surveillance scenarios involving dense crowds and complex activities.

### 3.1. Data Preprocessing and Identity-Aware Graph Construction

**Figure 2:**
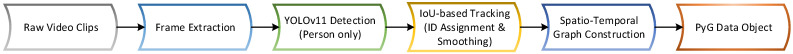
Data Processing Pipeline for Spatiotemporal Relationship Modeling of Human Interactions in Videos.

To enable spatiotemporal relationship modeling of human interactions in videos, we design a processing pipeline based on the raw video data, consisting of data cleaning, frame extraction, object detection, cross-frame identity tracking, and spatiotemporal graph construction. The overall workflow is illustrated in Figure 2.

The entire data preprocessing pipeline begins with Raw Video Clips, followed by Frame Extraction to obtain continuous frame sequences. Next, the YOLOv11x model is employed for object detection, retaining only bounding boxes corresponding to the person category. On this basis, a lightweight tracker with IoU-based matching is applied for ID Assignment & Trajectory Smoothing, ensuring identity consistency of the same individual across frames. Additionally, the tracker performs real-time updates, and a frame-level NMS threshold of 0.3 is applied to filter low-confidence detections.

Following this, person instances with stable IDs are mapped to graph nodes, and a Spatio-Temporal Graph is constructed based on spatial proximity and temporal continuity. The graph incorporates edges that represent person-person interactions within the same frame and between consecutive frames, capturing the dynamic relations. This graph is then formatted into a PyTorch Geometric (PyG) Data object (PyG v2.6.1), providing structured representations for subsequent graph neural network modeling. This structure enables the efficient modeling of interactions, leveraging both spatial and temporal features for robust identity tracking.

#### 3.1.1. Video Preprocessing and High-Precision Person Detection

To ensure the validity of the input data and the stability of subsequent modeling, the raw video data are first preprocessed by removing clips that are irrelevant to the research task or severely degraded in quality. The remaining videos are then decomposed into continuous frame sequences at a fixed frame rate.

In the detection stage, we adopt YOLO11x.pt as the object detection model to perform inference on each video frame, retaining only the detections classified as person. YOLO11x represents the largest variant within the YOLOv11 [25] family, containing 56.9M parameters and requiring 194.9B FLOPs, with an mAP50–95 of 54.7 on the COCO dataset—achieving higher detection accuracy than other YOLOv11 versions. Although computationally more expensive, its deeper and wider network architecture combined with multi-scale feature fusion enables superior robustness in small-object detection, occlusion handling, and crowded scenes. The detection results are output in the form of pixel coordinates (x_1_, y_1_, x_2_, y_2_), which are further converted into normalized center coordinates (c_x_, c_y_) and width–height (w, h) within the [0, 1] range to ensure consistent input scales. Leveraging the high-precision detection capability of YOLO11x.pt together with a stable target assignment mechanism, the model can assign consistent ID labels to the same individuals across frames during inference, thereby achieving preliminary cross-frame identity association while effectively reducing both missed detections and false positives.

However, in crowded scenes or cases where individuals are in close proximity, the pretrained YOLO model may mistakenly merge multiple adjacent persons into a single bounding box, leading to missed detections or identity confusion. To mitigate such issues, this study further incorporates the following optimization strategies during the preprocessing stage:

(1) Scale Filtering: Remove detection boxes that are excessively small or have abnormal aspect ratios to reduce noise interference. (2) Multi-Scale/High-Resolution Inference: Enhance detection accuracy in crowded scenarios and mitigate the merging of adjacent targets. (3) NMS Threshold Adjustment: Lower the non-maximum suppression threshold appropriately to preserve independent detections of closely positioned individuals.

**Figure 3:**
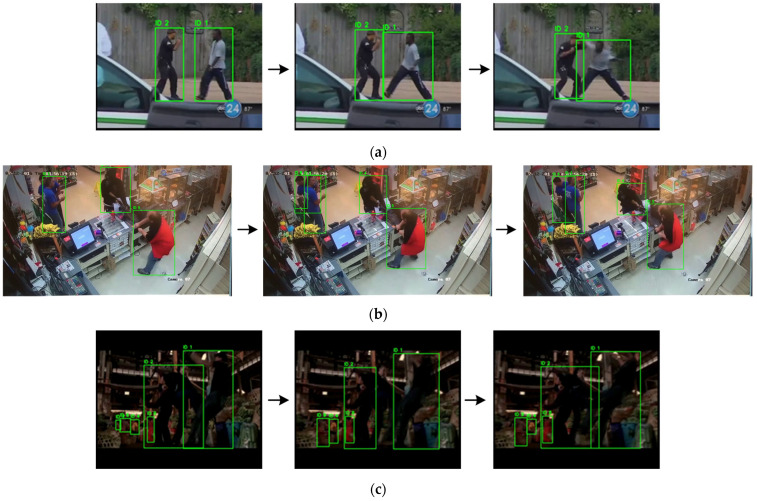
YOLO-based person detection and ID tracking across three scenes (**a**) outdoor street; (**b**) indoor surveillance; (**c**) low-light industrial setting.

As shown in Figure 3, the three scenes are (a) outdoor street, (b) indoor surveillance, and (c) low-light industrial environment. Frames in each row are arranged in temporal order from left to right. The YOLO11x.pt model accurately detects persons and maintains consistent track IDs across frames, providing a reliable basis for subsequent identity-aware graph construction.

#### 3.1.2. Identity Association and Graph Construction

After completing frame-level person detection and preliminary ID assignment during the data preprocessing stage, this study further incorporates a lightweight tracking strategy based on Intersection over Union (IoU) matching to enhance the continuity and robustness of cross-frame identity association. Let the detected bounding box of a person in the current frame be denoted as Ba, and the most recent position of an existing trajectory be denoted as Bb. The IoU is defined as

(1) $${\mathrm{IoU}(B}_{a}{,B}_{b})=\frac{\left|B_{a}\cap B_{b}\right|}{\left|B_{a}\cup B_{b}\right|}$$

In the figure, $\left|B_{a}\cap B_{b}\right|$ denotes the area of intersection between the two bounding boxes, while $\left|B_{a}\cup B_{b}\right|$ represents the area of their union. If the matching score exceeds the predefined threshold $\tau_{iou}$ (set to 0.5 in our experiments), the detection result is assigned to the corresponding trajectory, and its position and temporal information are updated. Unmatched detections will generate new trajectories with new IDs, whereas unmatched historical trajectories are removed once their consecutive missing frames exceed the upper limit max_lost (set to 10 frames in our experiments). This strategy, with low computational overhead, effectively maintains the stability of identity labels in short sequences and tolerates transient detection failures caused by occlusion or missed detections, thereby reducing identity loss.

When constructing the Identity-aware Spatiotemporal Graph, each person instance with a stable ID is modeled as a node, and its node feature vector is defined as

(2) $$x_{i}=[c_{x}^{\left(i\right)},c_{y}^{\left(i\right)},w^{\left(i\right)},h^{\left(i\right)},\frac{t_{i}}{T_{\max}}]$$

Here, $\left(c_{x}^{\left(i\right)},c_{y}^{\left(i\right)}\right)$ denote the center coordinates of node i, while $w^{\left(i\right)}$ and $h^{\left(i\right)}$ represent its width and height (all normalized to the range [0, 1]). $t_{i}$ is the frame index, and $T_{\max}$ is the maximum frame index of the video, used for temporal normalization. This design not only preserves the individual’s spatial positional information but also explicitly encodes temporal information into the node features, enabling downstream graph neural networks to jointly leverage spatial and temporal dimensions.

The rules for edge construction are divided into two categories:

1. Temporal edges

(3) $$e_{ij}^{time}=\left\{\begin{array}{c} 1,ifID_{i}=ID_{j}\wedge 0<|t_{i}-t_{j}|\leq T_{\omega }\\ 0,otherwise \end{array}\right\}$$

Here, $T_{\omega }$ denotes the temporal window size (set to 2 frames in our experiments), which is used to reflect the continuity of the motion trajectory of the same individual within a short time span.

2 Spatial edges

(4) $$e_{ij}^{space}\left\{\begin{array}{c} 1,ift_{i}=t_{j}\wedge ||P_{i}-P_{j}|{|}_{2}<d_{th}\\ 0,otherwise \end{array}\right.$$

Here, $P_{i}=(c_{x}^{\left(i\right)},c_{y}^{\left(i\right)})$ denotes the center coordinates of individual i, and $d_{th}$ represents the spatial distance threshold (set to 0.3 in our experiments after normalization), which is used to describe local interaction relationships among individuals within the same frame.

Each edge is associated with an edge feature vector, defined as

(5) $$a_{ij}=[\frac{\left|t_{i}-t_{j}\right|}{\alpha_{t}},\frac{||P_{i}-P_{j}|{|}_{2}}{\alpha_{d}}]$$

Here, $\alpha_{t}=5.0$ and $\alpha_{d}=300.0$ are normalization constants for temporal intervals and spatial distances, respectively. This design not only preserves the original temporal–spatial information but also maps the feature values into a numerical range suitable for graph neural network inputs. If no nodes in the video meet the specified conditions, a single-node graph with zero-valued features is generated to avoid structural deficiencies in downstream modeling.

**Figure 4:**
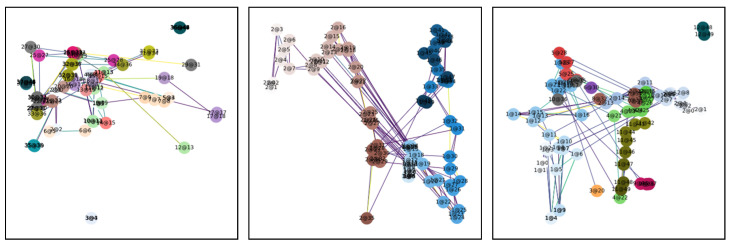
Identity-Aware Graphs from Different Videos.

To provide an intuitive illustration of the constructed spatiotemporal graph structure, we use NetworkX for visualizing nodes and edges. Node colors are assigned based on individual IDs, with brightness gradually deepening as time progresses, reflecting the passage of time in a static graph. Node labels are formatted as “ID@Frame,” capturing the corresponding trajectory patterns. In Figure 4, nodes of different colors represent unique individual identities, and the edges signify their spatial or temporal associations. Edges are constructed based on the spatial and temporal proximity between nodes. Specifically, if two nodes belong to the same individual (i.e., they share the same ID) and their time difference is within a specified temporal window, an edge is formed. Additionally, spatial edges are drawn when the Euclidean distance between two nodes is smaller than a spatial threshold. The color of the edges varies depending on the distance between nodes. Edges representing closer relationships (either spatial or temporal) are darker, while edges representing more distant relationships are lighter, creating a visual representation of the strength of the connections. Trajectories formed by same-colored nodes indicate the movement paths of individuals, while the temporal progression is conveyed through the darkening of node brightness. Three sets of visualizations showcase identity-aware graph structures in varying scenarios, from sparse distributions to dense interactions. These visualizations maintain the stability of individual IDs and ensure the rationality of edge connections, demonstrating the method’s adaptability across different environments.

Finally, the identity-aware graph is stored in the form of a PyTorch Geometric Data object, which includes the node feature matrix, edge indices, edge features, ID list, and frame indices, serving as the direct input for downstream spatiotemporal graph neural networks.

### 3.2. Architecture Choice

The architecture of IDG-ViolenceNet combines 3D-CNN with GINEConv to effectively capture both spatial and temporal features in video data.

3D-CNN: 3D-CNN are widely used for processing video data because they can capture both spatial and temporal features by applying convolutional filters across both the spatial dimensions (height and width of the frame) and the temporal dimension (time). In our model, the 3D-CNN layers are responsible for extracting local spatiotemporal features from video frames. This allows the model to detect motion, objects, and other important temporal dynamics within the video. 3D-CNN are ideal for tasks like action recognition or violence detection, where understanding both the space and time in the video is crucial.

GINEConv: In addition to spatial and temporal feature extraction, understanding the interactions between individuals in the video is essential for detecting violent behavior. For this purpose, we use GINEConv, a type of Graph Neural Network. GINEConv is used to model the relationships between individuals in the video by representing each person as a node in a graph, with edges representing interactions between them. The GINEConv layer aggregates information from neighboring nodes (individuals), allowing the model to capture the dynamic interactions between people over time, which is essential for detecting violence in scenes with multiple people.

By combining 3D-CNN and GINEConv, our model benefits from the strengths of both architectures: 3D-CNN for extracting local spatiotemporal features and GINEConv for modeling complex interactions between individuals. This hybrid architecture is particularly effective for video violence detection, as it can learn both individual actions and the relationships between individuals in the video, which is crucial for detecting violent behavior in dynamic and crowded scenes.

### 3.3. Hyperparameter Selection

In our experiments, we focused on tuning two critical hyperparameters: the learning rate and the batch size, as these significantly affect model performance and training stability.

Learning Rate: We set the learning rate to 0.001, which is a commonly used value for the Adam optimizer. A learning rate of 0.001 was tested and found to provide stable training without causing large fluctuations in the loss function. During preliminary experiments, we tested learning rates ranging from 0.0001 to 0.1. A learning rate of 0.001 consistently provided the best validation performance, ensuring the model converged without overshooting the optimal solution. Higher learning rates (e.g., 0.01 or 0.1) led to instability in training, where the model’s accuracy fluctuated significantly, and convergence was not achieved. Lower learning rates (e.g., 0.0001) resulted in slower convergence, leading to extended training times without a noticeable improvement in validation accuracy. Based on these empirical results, we selected 0.001 as the optimal learning rate to balance training speed and model performance.

Batch Size: Due to hardware configuration limitations, we experimented with three different batch sizes: 8, 16, and 32. A batch size of 16 was found to provide the best performance, striking the right balance between training time and model accuracy. Smaller batch sizes, such as 8, led to noisier gradient updates and slower convergence, while batch size 16 resulted in faster convergence and the best validation accuracy. We tested various batch sizes during preliminary experiments, and batch size 16 consistently performed better on the validation set, providing the best trade-off between computational efficiency and model generalization.

These hyperparameters were chosen based on a combination of empirical results and prior research, with the goal of providing stable training while achieving optimal model performance. The validation results confirmed that these settings were optimal for our task, leading to faster convergence, better generalization, and improved overall performance.

### 3.4. Multi-Branch Spatiotemporal Modeling Network

**Figure 5:**
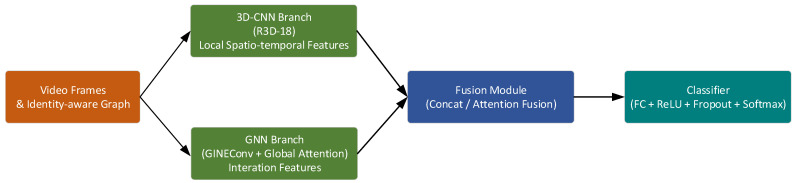
Multi-Branch Video Modeling Framework.

To simultaneously capture local spatiotemporal variation patterns and global interaction structures in videos, we propose a multi-branch video modeling framework that integrates 3D-CNNs and GNNs, as illustrated in Figure 5. The framework consists of two parallel branches:

In the spatiotemporal feature extraction branch, we employ the R3D-18-based 3D Convolutional network [26], which is designed to model local motion patterns and capture spatiotemporal dependencies within short-term frame sequences. Specifically, the input sequence length is set to 8 frames with a fixed resolution. All convolutional layers adopt a kernel size of (3, 3, 3), with the channel dimension starting from 64 and progressively increasing within the residual blocks. ReLU is used as the activation function, global average pooling is applied for feature aggregation, and a Dropout layer is inserted before the fully connected layer to alleviate overfitting.

In the identity-aware graph feature modeling branch, we adopt a GINEConv-based Graph Neural Network [27], which is designed to model the spatial relationships among multiple individuals and capture cross-frame interaction dependencies within a video. Each node is represented by a 5-dimensional feature vector (center coordinates, width, height, and normalized timestamp), while each edge is described by a 2-dimensional feature vector (normalized temporal interval and spatial distance). Node and edge features are projected into a 128-dimensional hidden space through fully connected layers. One-hop neighborhood aggregation is then performed, followed by Global Attention Pooling for feature aggregation. To enhance generalization, batch normalization and Dropout are applied after each convolutional layer.

During training, the batch size is set to 16. The learning rate is selected from {0.0005, 0.001, 0.0015}, with 0.001 yielding the best performance. The Adam optimizer is used in conjunction with a ReduceLROnPlateau scheduler, and the loss function is Cross-Entropy Loss. Early stopping is employed, halting training if validation performance shows no improvement for more than 5 consecutive epochs.

Figure 5 illustrates the overall structure of the proposed multi-branch spatiotemporal modeling network: the inputs on the left consist of video frames and the identity-aware graph, which are fed into the R3D-18 branch and the GNN branch, respectively, for feature extraction; the fusion module integrates the features from both modalities; and finally, the classifier outputs the recognition results.

### 3.5. 3D-CNN Branch: Local Spatiotemporal Feature Extraction

To capture local motion patterns within video clips, we adopt R3D-18 as the backbone for convolutional feature extraction. Structurally, R3D-18 inherits the four residual stages of ResNet-18 [28] (Conv1, Conv2_x, Conv3_x, Conv4_x) while extending the convolutional kernels from two dimensions ($k_{h}\times k_{w}$) to three dimensions ($k_{t}\times k_{h}\times k_{w}$) enabling the model to perform feature modeling simultaneously along the temporal, vertical, and horizontal dimensions.

At the input stage, to ensure stability in temporal modeling, the video frame sequence is uniformly sampled to a fixed length of T=8 frames, yielding local cropped images of detected persons. Each frame is cropped using the detection bounding box and resized to 112×112 pixels, forming the input tensor:

(6) $$X\in R^{B\times 3\times T\times 112\times 112}$$

Here, B denotes the batch size. The 3D convolution operation in R3D-18 is formulated as

(7) $$Y_{t,h,w}=\sum_{△t=0}^{k_{t}-1}\sum_{△h=0}^{k_{s}-1}\sum_{△w=0}^{k_{s}-1}W_{△t,△h,△w}\cdot X_{t+△t,h+△h,w+△w}$$

In our experiments, both the temporal kernel size $k_{t}$ and the spatial kernel size $k_{s}$ are set to 3. The stride is set to 1 along the temporal dimension and 2 along the spatial dimensions (for spatial downsampling). The network weights are initialized from a model pretrained on the Kinetics-400 dataset, leveraging its rich and generalizable motion features.

The global feature produced by R3D-18 has the shape $\left[B,512,1,1,1\right]$ and is projected by a fully connected layer (cnn_fc) to a $d_{cnn}=256$-dimensional vector, yielding the local spatiotemporal representation $f_{3D}$. This representation is highly sensitive to short-term action variations (e.g., shoving, punching, kicking), making it particularly well-suited for scenarios such as violence detection that require capturing rapid movements.

### 3.6. GNN Branch: Modeling Spatial Interaction Structures

To explicitly model the spatiotemporal interactions among multiple individuals in videos, we employ GINEConv [29] (Graph Isomorphism Network with Edge features) to perform graph convolution on the identity-aware graph. GINEConv is an effective Graph Neural Network (GNN) architecture that can handle graph data with edge features, making it especially suitable for graph structures enriched with edge features, such as relationships or interactions between individuals. In this model, each node represents a person instance, and each edge represents the spatiotemporal interactions between them. The GINEConv layer allows for the capture of spatiotemporal dependencies and dynamic interactions between individuals, providing more efficient modeling for multi-person tracking and recognition in videos. In the specific implementation, GINEConv uses two fully connected layers (MLP) to construct the convolution operation, updating both node features and edge features through this network. By applying nonlinear activation (ReLU) to the output of each layer, and aggregating the graph nodes through Global Attention pooling, the model is able to effectively capture complex spatiotemporal interaction patterns between individuals in the video.

Input Graph Structure:

① Node features $X_{v}\in R^{d_{x}}$: composed of the center coordinates $\left(c_{x},c_{y}\right)$, width–height $\left(w,h\right)$, and the normalized timestamp $t_{norm}$.
② Edge features $e_{uv}\in R^{d_{e}}$: composed of the normalized temporal interval and normalized Euclidean distance, which, respectively, represent the temporal relationships and spatial proximity between individuals.

First, two independent linear transformations are applied to project both node and edge features into a unified hidden dimension of $d_{h}=128$:

(8) $$h_{v}^{\left(0\right)}{=W}_{x}x_{v}$$

(9) $$z_{uv}=W_{e}e_{uv}$$

Subsequently, during the message-passing phase, GINEConv updates node representations by jointly leveraging both node features and edge features:

(10) $$h_{v}^{\left(l+1\right)}=MLP^{\left(l\right)}((1+\varepsilon^{\left(l\right)})h_{v}^{\left(l\right)}+\sum_{u\in Ν(v)}\sigma (h_{u}^{\left(l\right)}+z_{uv}))$$

Here, Ν(v) denotes the set of neighbors of node v, σ is the ReLU activation function, and $\varepsilon^{\left(l\right)}$ is a learnable constant. After multiple layers of graph convolutions, the local information of nodes is progressively aggregated into a global graph-level representation.

Finally, Global Attention Pooling is applied to aggregate node features with attention weights into a video-level representation, producing a global interaction feature vector $f_{GNN}$ of dimension $d_{gnn}=128$. This vector explicitly characterizes the interaction patterns and spatiotemporal structural relationships among multiple individuals, providing strong feature support for distinguishing different types of group behaviors (e.g., confrontation, cooperation).

### 3.7. Multimodal Feature Fusion Module

Before the classification stage, we introduce a multimodal feature fusion module to combine the complementary strengths of two modalities: the local spatiotemporal visual modality (extracted by R3D-18, representing appearance and short-term motion patterns in frame sequences) and the structured interaction modality (modeled by the GNN on the identity-aware graph, capturing spatial relationships and temporal dependencies among individuals). Since these modalities differ fundamentally in data representation and feature space, they can be regarded as heterogeneous modalities. By employing concatenation or attention-based weighted fusion, the model can adaptively integrate fine-grained motion cues with global interaction structures, thereby significantly enhancing the robustness and generalization ability of violence recognition. The fusion strategies are divided into two categories:

1. Concatenation Fusion

When attention-based fusion is not applied, the feature vector $f_{3D}\in R^{d_{cnn}}$ from the CNN branch and the feature vector $f_{GNN}\in R^{d_{gnn}}$ from the GNN branch are directly concatenated along the channel dimension:

(11) $$f_{fused}=[f_{3D}||f_{GNN}]\in R^{d_{cnn}+d_{gnn}}$$

This approach preserves the complete information from both modalities and is suitable for scenarios where the feature dimensions are relatively small and computational resources are sufficient.

2. Attention Fusion

When attention-based fusion is enabled, the features from both modalities are first linearly projected to a common dimension $d_{c}=\min(d_{cnn},d_{gnn})$:

(12) $${\hat{f}}_{3D}=W_{cnn}f_{3D}$$

(13) $${\hat{f}}_{GNN}=W_{gnn}f_{GNN}$$

Then, the aligned features are concatenated and fed into the attention weighting network to generate modality weights $w=[w_{cnn},w_{gnn}]$:

(14) $$w=Softmax(W_{2}\sigma (W_{1}[{\hat{f}}_{3D}||{\hat{f}}_{GNN}]))$$

The final fused feature is obtained by the weighted summation of the two modalities:

(15) $$f_{fused}=w_{cnn}\times {\hat{f}}_{3D}+w_{gnn}\times {\hat{f}}_{GNN}$$

Attention-based fusion can adaptively adjust the contribution ratio of the two modalities according to the specific content of the input video. For example, in interaction-intensive scenarios, the weight of the GNN features may be higher, whereas in segments with pronounced rapid movements, the weight of the CNN features may dominate.

Regardless of the fusion strategy adopted, the final fused vector is fed into a fully connected classifier

FC→ReLU→Dropout(p=0.5)→Softmax

to output the class probability distribution of the video (violence/non-violence). This fusion design ensures that the local motion modeling capability of the CNN and the global structural modeling capability of the GNN complement each other, thereby enhancing the overall performance of video behavior recognition.

## 4. Experiments

### 4.1. Experimental Setup and Datasets

The experiments were conducted on a Windows 10 operating system with an Intel(R) Xeon(R) W-2275 @ 3.30GHz CPU, and accelerated using an NVIDIA RTX A4000 GPU. Python 3.8 was used as the programming language, while model construction, training, and optimization were implemented with the PyTorch deep learning framework and the PyTorch Geometric (PyG) library for graph neural networks.

To ensure the reliability and validity of our research results, we evaluate the proposed model on three widely used benchmark datasets: Hockey Fight, Movies Fight, and RWF-2000. The Hockey Fight dataset, constructed by Nievas et al. [30], consists of 500 violent and 500 non-violent short video clips, with each video containing an average of 41 frames. The Movies Fight dataset, also created by Nievas et al. [30], contains 201 videos—100 violent and 101 non-violent—each lasting approximately 1.6–2 s with around 50 frames on average. The RWF-2000 dataset, introduced by Cheng et al. [31], is a large-scale violence detection dataset built from real-world surveillance footage. It includes 1000 violent and 1000 non-violent clips, each about 5 s in length and averaging 150 frames. Compared with the other two datasets, RWF-2000 is more challenging, as it incorporates complex factors such as low illumination, blur, and occlusion, making it highly representative of real-world scenarios.

For all datasets, we adopt a 6:2:2 split into training, validation, and test sets using a video-level, class-stratified protocol to prevent data leakage. Specifically, all clips or frames originating from the same source video or camera scene are assigned exclusively to a single partition. After splitting, identity-aware graphs are constructed for each video, ensuring that tracking identities or near-duplicate frames do not cross between partitions.

### 4.2. Evaluation Metrics

To comprehensively evaluate the classification performance of the proposed method on video violence detection, we adopt several commonly used metrics, including Accuracy, Precision, Recall, F1-Score, and the Area Under the Receiver Operating Characteristic Curve (AUC). In the evaluation process, violent videos (fight) are defined as the positive class, while non-violent videos (nonfight) are defined as the negative class.

Here, TP (True Positive) denotes the number of positive samples correctly classified as positive; TN (True Negative) denotes the number of negative samples correctly classified as negative; FP (False Positive) denotes the number of negative samples incorrectly classified as positive; and FN (False Negative) denotes the number of positive samples incorrectly classified as negative. The calculation formulas for each metric are as follows:

(16) $$A\mathrm{ccuracy}=\frac{TP+TN}{TP+TN+FP+FN}$$

(17) $$Precision=\frac{\mathrm{TP}}{\mathrm{FP}+\mathrm{TP}}$$

(18) $$F1=\frac{2\times \mathrm{Precision}\times \mathrm{Recall}}{\mathrm{Precision}+\mathrm{Recall}}$$

(19) $$f_{fused}=[f_{3D}||f_{GNN}]\in R^{d_{cnn}+d_{gnn}}$$

Accuracy reflects the overall correctness of the model’s predictions; Precision measures the proportion of samples predicted as positive that are truly positive; Recall measures the proportion of positive samples that are correctly identified; and the F1-Score, as the harmonic mean of Precision and Recall, is more suitable for evaluating models under imbalanced class distributions.

In addition, to evaluate the model’s discriminative ability under different classification thresholds, this paper calculates the AUC. Specifically, in implementation, the value of the positive-class channel output from the Softmax function $p\left(y=1|x\right)$ is taken as the discriminant score. By traversing all possible thresholds, the ROC curve is plotted, and the area under the curve is computed as the AUC value. AUC values closer to 1 indicate stronger separability of positive and negative classes by the model.

### 4.3. Experimental Results and Analysis

To comprehensively evaluate the effectiveness and generalization capability of the proposed method, we conducted systematic experiments on three public violence recognition datasets: Hockey Fight, Movies Fight, and RWF-2000. The compared baselines include traditional optical flow-based methods (Vif), temporal modeling approaches (ECO), graph convolutional networks (DGCNN), lightweight spatiotemporal modeling (MobileNet-TSM), and Transformer-based methods (MoEViT). All methods were tested under the same hardware setup and dataset partitioning strategy to ensure fair and comparable results.

Table 1 presents the classification accuracy comparison of different methods on the three datasets. It can be observed that the proposed IDG-ViolenceNet achieves the highest accuracies of 97.5% and 99.5% on the Hockey Fight and Movies Fight datasets, respectively, and reaches 89.4% on the RWF-2000 dataset. While maintaining state-of-the-art accuracy, the model also demonstrates strong adaptability to challenging surveillance scenarios such as low resolution, viewpoint variation, and crowded scenes. These results indicate that the proposed method exhibits robust performance and strong generalization capability across diverse violence recognition tasks.

We conducted a systematic evaluation of IDG-ViolenceNet on three benchmarks—HockeyFight, MoviesFight, and RWF-2000—using Accuracy, Precision, Recall, F1-Score, and AUC. All results are averaged over five random seeds and reported as mean ± std. Accuracy measures overall correctness; Precision and Recall characterize exactness and coverage for the positive class; F1 balances Precision and Recall; and AUC assesses discriminative ability across thresholds. As shown in Table 2, the model performs best on Movie Fight. Overall, training time increases with dataset complexity, while inference time remains largely stable. Taken together, the five-seed mean ± std results demonstrate high accuracy on constrained datasets and strong robustness and adaptability in more realistic surveillance scenarios.

**Figure 6:**
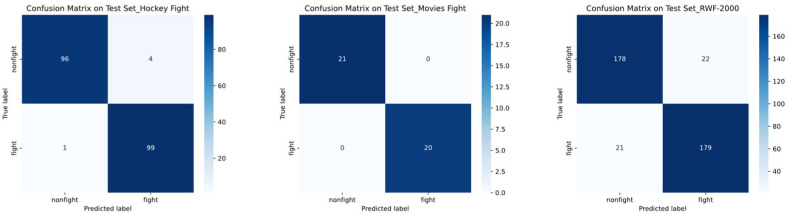
Confusion Matrices for Hockey Fight, Movies Fight, and RWF-2000 Datasets.

In addition to the quantitative metrics, we further examined the model’s performance through confusion matrices on the three benchmark datasets, as illustrated in Figure 6. These matrices provide a more fine-grained view of the classification results. For the Hockey Fight dataset, the model achieved an accuracy of 97.5%, with only a few samples misclassified. On the Movies Fight dataset, the model achieved 100% accuracy on the shown test split (21/21 and 20/20 correctly classified), while the average performance across multiple random runs or cross-validation folds was 99.50 ± 0.50%. In contrast, for the more challenging RWF-2000 dataset, the model achieved an accuracy of 89.4%, where most errors occurred in mislabeling “nonfight” actions as “fight.” This analysis highlights not only the robustness of the proposed approach on simpler datasets but also its limitations and generalization ability in more complex, real-world scenarios.

**Figure 7:**
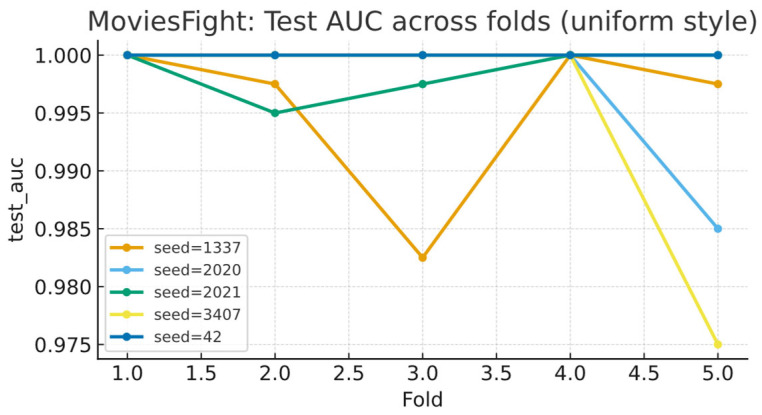
Per-seed five-fold test AUCs on the MoviesFight dataset.

To systematically evaluate stability and generalization under small test folds (≈40–41), we use five random seeds (42, 1337, 2020, 2021, 3407) and perform five-fold cross-validation for each seed, yielding 25 independent test folds in total; the evaluation metrics include accuracy (ACC) and AUC. The results show that across all 25 test folds, MoviesFight achieves a mean ACC of 99.5%, with a minimum/maximum of 97.50%/100.00%; among these, 18/25 folds reach 100%, indicating high consistency and stability across seeds and folds. In terms of AUC, the five seed-specific five-fold curves remain close to 1.0 overall, with only slight dips in a few folds (see Figure 7), suggesting that local difficulty variations do not alter the overall high and robust discriminative performance.

### 4.4. Ablation Study

To comprehensively evaluate the contribution of each component to the model’s performance and its applicability across different scenarios, we conducted ablation experiments on all three datasets; the results are summarized in Table 3, Table 4 and Table 5. Under identical training/validation splits, optimizer settings, and hyperparameters, we varied only the feature extraction and fusion strategies, considering the following four variants:

(1) CNN_Only: prediction using only CNN features; (2) GNN_Only: prediction using only GNN structural information; (3) CNN+GNN (Concat): concatenation of CNN and GNN features followed by the classification head; and (4) CNN+GNN (Attn): cross-modal/cross-domain fusion using an attention mechanism (our full method).

All experiments are compared in terms of Accuracy, F1-Score, and AUC, in order to validate the stability and generalization ability of each component under different data distributions and task scenarios.

From the ablation results above, it can be observed that the overall performance of IDG-ViolenceNet relies on the complementary characteristics of the CNN and GNN branches. Using only the CNN branch (CNN_Only) achieves relatively high Accuracy and F1-Score across all three datasets, indicating that the 3D-CNN is highly effective in capturing short-term local motion features. However, the performance of GNN_Only is generally lower than that of CNN_Only, with particularly significant gaps on the Hockey Fight and Movies Fight datasets. This reflects the limited discriminative power of relying solely on structured interaction features when fine-grained visual information is absent. The concatenation of features from both branches (CNN+GNN Concat) yields performance improvements on certain datasets, validating the complementarity between local visual features and global interaction features. With the further introduction of attention-based weighted fusion (CNN+GNN Attn), the model achieves the best results in terms of Accuracy, F1-Score, and AUC. Notably, it attains 100% Accuracy and F1-Score on the Movies Fight dataset, demonstrating that the attention mechanism can adaptively assign importance between the two modalities depending on the scene, thereby maximizing fusion effectiveness. Overall, the ablation study results strongly validate the critical roles of identity-aware graph construction, dual-branch feature extraction, and the attention-based fusion module in enhancing both the accuracy and robustness of violence detection under complex scenarios.

### 4.5. Limitations and Future Work

The limitations of this study are as follows: Our current pipeline does not yet support reliable edge-level attribution, and the available datasets lack edge-level ground truth; therefore, the present visualizations are intended to illustrate graph construction and edge-weight distributions rather than single-edge causality. In addition, we have not yet built our own multi-scene violence video dataset, which constrains extrapolation to complex real-world settings. In future work, we will make these limitations explicit and systematically incorporate edge-level explanation methods (masking-based perturbation analyses, GNNExplainer/PGExplainer, and structural ablations on spatial vs. temporal edges), accompanied by randomized and stability checks and metrics such as deletion/insertion curves; construct small-scale edge-labeled or controllable synthetic benchmarks for calibration; and curate and release a diversified violence video dataset (covering viewpoint, illumination, occlusion, group size, and action intensity) with fixed splits and baseline code. All releases will adhere to compliance and privacy requirements, with the aim of improving the reliability of explanations, the robustness of results, and the reproducibility of the research.

## 5. Conclusions

The proposed IDG-ViolenceNet model introduces an identity-aware graph construction mechanism combined with a dual-branch 3D-CNN–GNN feature extraction framework, enabling collaborative modeling of multi-person spatial interactions and local spatiotemporal motion patterns in videos. Experimental results demonstrate that the method consistently outperforms mainstream approaches across multiple public datasets, particularly showing strong generalization and robustness in surveillance scenarios characterized by dense crowds, severe occlusions, and complex actions. Further ablation studies validate the critical role of cross-frame identity-preserving graph modeling and cross-modal feature fusion in improving detection accuracy. Future work will focus on two main directions: (1) further optimizing person detection and tracking algorithms to enhance identity association stability under ultra-dense crowds and extreme occlusion conditions and (2) exploring the integration of adaptive spatiotemporal attention mechanisms and multimodal information (e.g., audio signals and scene semantics) to improve adaptability to more complex forms of violent behavior and cross-domain datasets. Overall, this study not only provides a feasible paradigm for deep fusion of structured and visual features but also offers strong technical support for implementing real-time early-warning modules in intelligent security systems.

## Figures and Tables

**Table 1:** Comparison of Accuracy (%) of Different Methods on Three Datasets.

Methods	Hockey Fight	Movies Fight	RWF-2000
Vif [15]	81.6	—	80.9
ECO [32]	94.0	96.3	83.7
DGCNN [33]	90.2	92.6	80.6
MobileNet-TSM [34]	97.5	—	87.75
MoEViT [35]	—	—	92.4
STIG-Net [24]	95.5	98.3	85.1
IDG-ViolenceNet	97.5	99.5	89.4

**Table 2:** Performance Comparison of the Proposed Method on Different Datasets.

Datasets	Accuracy (%)	Precision (%)	Recall (%)	F1-Score (%)	AUC	epoch_train_time_mean (s)	epoch_test_time_mean (s)
Hockey Fight	97.50 ± 2.35	97.03 ± 3.04	99.00 ± 3.21	98.01 ± 2.92	0.99 ± 0.024	121.42 ± 2.52	12.22 ± 0.10
Movie Fight	99.50 ± 0.85	99.80 ± 0.50	99.60 ± 0.70	99.70 ± 0.60	0.996 ± 0.008	13.09 ± 0.77	12.31 ± 0.29
RWF-2000	89.40 ± 2.65	90.00 ± 2.70	91.04 ± 2.98	89.9 ± 2.24	0.91 ± 0.018	193.39 ± 69.75	12.26 ± 0.52

**Table 3:** Ablation Study Results on the Hockey Fight Dataset.

Methods	Acc(%)	F1(%)	AUC
CNN_Only	94.00	94.17	0.99
GNN_Only	84.00	85.05	0.92
CNN + GNN (Concat)	97.00	97.06	0.99
CNN + GNN (Attn)	97.50	97.54	0.99

**Table 4:** Ablation Study Results on the Movies Fight Dataset.

Methods	Acc(%)	F1(%)	AUC
CNN_Only	97.56	97.56	0.99
GNN_Only	92.68	92.68	0.97
CNN + GNN (Concat)	97.56	97.56	0.99
CNN + GNN (Attn)	99.50	99.70	0.99

**Table 5:** Ablation Study Results on the RWF-2000 Dataset.

Methods	Acc(%)	F1(%)	AUC
CNN_Only	78.25	79.04	0.87
GNN_Only	76.50	78.54	0.86
CNN + GNN (Concat)	75.75	72.98	0.86
CNN + GNN (Attn)	89.4	89.9	0.91

## Data Availability

The datasets used in this study are publicly available. The RWF-2000 dataset can be accessed at https://paperswithcode.com/dataset/rwf-2000 (accessed on 2 March 2024.), the Hockey Fight dataset at https://academictorrents.com/details/38d9ed996a5a75a039b84cf8a137be794e7cee89 (accessed on 28 May 2024), and the Movies Fight dataset at https://www.kaggle.com/datasets/naveenk903/movies-fight-detection-dataset (accessed on 28 May 2024). All datasets were used in accordance with their respective licenses.

## References

[1] Pandey B., Sinha U., Nagwanshi K.K. (2025). A multi-stream framework using spatial–temporal collaboration learning networks for violence and non-violence classification in complex video environments. Int. J. Mach. Learn. Cybern..

[2] Kim H., Lee B.S., Shin W.-Y., Lim S. (2022). Graph anomaly detection with graph neural networks: Current status and challenges. IEEE Access.

[3] Ahmad T., Jin L., Zhang X., Lai S., Tang G., Lin L. (2021). Graph convolutional neural network for human action recognition: A comprehensive survey. IEEE Trans. Artif. Intell..

[4] Patel D., Sarlati S., Martin-Tuite P., Feler J., Chehab L., Texada M., Marquez R., Orellana F.J., Henderson T.L., Nwabuo A. (2020). Designing an information and communications technology tool with and for victims of violence and their case managers in San Francisco: Human-centered design study. JMIR mHealth uHealth.

[5] Wang N., Zhu G., Zhang L., Shen P., Li H., Hua C. Spatio-temporal interaction graph parsing networks for human-object interaction recognition. Proceedings of the 29th ACM International Conference on Multimedia.

[6] Huang H., Zhou L., Zhang W., Corso J.J., Xu C. (2018). Dynamic graph modules for modeling object-object interactions in activity recognition. arXiv.

[7] Yun H., Ahn J., Kim M., Kim E.-S. Compositional video understanding with spatiotemporal structure-based transformers. Proceedings of the IEEE/CVF Conference on Computer Vision and Pattern Recognition.

[8] Ullah F.U.M., Ullah A., Muhammad K., Haq I.U., Baik S.W. (2019). Violence detection using spatiotemporal features with 3D convolutional neural network. Sensors.

[9] Abdali A.-M.R., Al-Tuma R.F. (2019). Robust real-time violence detection in video using cnn and lstm. Proceedings of the 2019 2nd Scientific Conference of Computer Sciences (SCCS).

[10] Patel M. (2021). Real-time violence detection using CNN-LSTM. arXiv.

[11] Khan M., Gueaieb W., Elsaddik A., De Masi G., Karray F. (2024). Graph-based knowledge driven approach for violence detection. IEEE Consum. Electron. Mag..

[12] Lai Z., Liang G., Zhou J., Kong H., Lu Y. (2024). A joint learning framework for optimal feature extraction and multi-class SVM. Inf. Sci..

[13] Salman H.A., Kalakech A., Steiti A. (2024). Random forest algorithm overview. Babylon. J. Mach. Learn..

[14] Schapire R.E. (2013). Explaining adaboost. Empirical Inference: Festschrift in Honor of Vladimir N. Vapnik.

[15] Hassner T., Itcher Y., Kliper-Gross O. (2012). Violent flows: Real-time detection of violent crowd behavior. Proceedings of the 2012 IEEE Computer Society Conference on Computer Vision and Pattern Recognition Workshops.

[16] Deniz O., Serrano I., Bueno G., Kim T.-K. (2014). Fast violence detection in video. Proceedings of the 2014 International Conference on Computer Vision Theory and Applications (VISAPP).

[17] Li J., Jiang X., Sun T., Xu K. (2022). Efficient violence detection using 3d convolutional neural networks. Proceedings of the 2019 16th IEEE International Conference on Advanced Video and Signal Based Surveillance (AVSS).

[18] Ghosh D.K., Chakrabarty A. (2022). Two-stream multi-dimensional convolutional network for real-time violence detection. arXiv.

[19] Negre P., Alonso R.S., González-Briones A., Prieto J., Rodríguez-González S. (2024). Literature Review of Deep-Learning-based detection of violence in video. Sensors.

[20] Kavathia A., Sayer S. (2024). Optimizing Violence Detection in Video Classification Accuracy through 3D Convolutional Neural Networks. arXiv.

[21] Scarselli F., Gori M., Tsoi A.C., Hagenbuchner M., Monfardini G. (2008). The graph neural network model. IEEE Trans. Neural Netw..

[22] Yang H., Ren Z., Yuan H., Wei W., Zhang Q., Zhang Z. (2022). Multi-scale and attention enhanced graph convolution network for skeleton-based violence action recognition. Front. Neurorobotics.

[23] Tian J., Li D. (2023). Video Violence Detection Method Based on Multi-Feature and Graph Convolutional Network. The International Conference on 3D Imaging Technologies.

[24] Lu X., Chen Y., Chen Y., Gao X., Yang T., Chen G. (2025). STIG-Net: A spatial–temporal interactive graph framework for recognizing violent behaviors in videos. Vis. Comput..

[25] He L.-H., Zhou Y.-Z., Liu L., Cao W., Ma J.-H. (2025). Research on object detection and recognition in remote sensing images based on YOLOv11. Sci. Rep..

[26] Byeon Y.-H., Kim D., Lee J., Kwak K.-C. (2021). Body and hand–object ROI-based behavior recognition using deep learning. Sensors.

[27] Yang Y., Zou D., He X. (2023). Graph neural network-based node deployment for throughput enhancement. IEEE Trans. Neural Netw. Learn. Syst..

[28] He K., Zhang X., Ren S., Sun J. Deep residual learning for image recognition. Proceedings of the IEEE Conference on Computer Vision and Pattern Recognition.

[29] Giuliari F., Skenderi G., Cristani M., Del Bue A. Spatial commonsense graph for object localisation in partial scenes. Proceedings of the IEEE/CVF Conference on Computer Vision and Pattern Recognition.

[30] Nievas E.B., Suarez O.D., Garcia G.B., Sukthankar R. (2011). Hockey fight detection dataset. Computer Analysis of Images and Patterns.

[31] Cheng M., Cai K., Li M. (2021). RWF-2000: An open large scale video database for violence detection. Proceedings of the 2020 25th International Conference on Pattern Recognition (ICPR).

[32] Zolfaghari M., Singh K., Brox T. Eco: Efficient convolutional network for online video understanding. Proceedings of the European Conference on Computer Vision (ECCV).

[33] Wang Y., Sun Y., Liu Z., Sarma S.E., Bronstein M.M., Solomon J.M. (2019). Dynamic graph cnn for learning on point clouds. ACM Trans. Graph..

[34] Zhang Y., Li Y., Guo S. (2022). Lightweight mobile network for real-time violence recognition. PLoS ONE.

[35] Mohammadi H., Nazerfard E., Firoozi T. (2023). Reinforcement Learning-based Mixture of Vision Transformers for Video Violence Recognition. arXiv.

